# Dimethyl 2,6-dimethyl-4-phenyl­pyridine-3,5-dicarboxyl­ate

**DOI:** 10.1107/S1600536811049865

**Published:** 2011-11-30

**Authors:** Mukut Gohain, Theunis J. Muller, Barend C. B. Bezuidenhoudt

**Affiliations:** a Department of Chemistry, University of the Free State, PO Box 339, Bloemfontein 9300, South Africa

## Abstract

In the title compound, C_17_H_17_NO_4_, the dihedral angle between the benzene and pyridine rings is 75.51 (4)°. The benzene and pyridine rings are both approximately planar (r.m.s. deviations of 0.0040 and 0.0083 Å, respectively), indicating that the pyridine N atom is not protonated. The crystal structure is stabilized by weak inter­molecular C—H⋯O and C—H⋯N inter­actions.

## Related literature

For the biological activity of pyridine derivatives, see: Lopez-Alarcon *et al.* (2004 ▶). For related structures, see: Rowan *et al.* (1996 ▶, 1997 ▶); Lou *et al.* (2010 ▶). For the sythesis, see: Debache *et al.* (2008 ▶). For the use of pyridine-type ligands in catalysis models, see: Roodt *et al.* (2011 ▶); van der Westhuizen *et al.* (2010 ▶) For standard bond lengths, see: Allen *et al.* (1987 ▶).
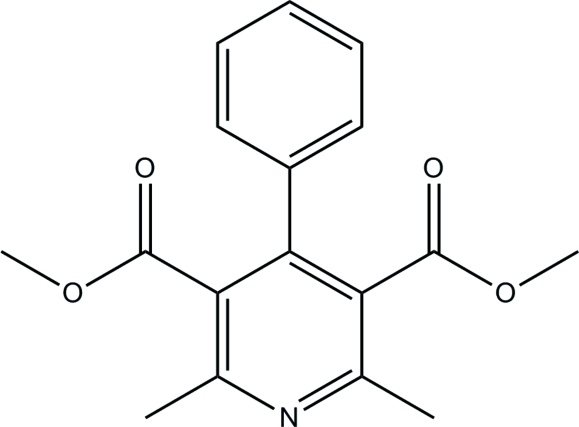


## Experimental

### 

#### Crystal data

C_17_H_17_NO_4_
*M* *_r_* = 299.32
Monoclinic, 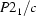

*a* = 16.0732 (4) Å
*b* = 7.2497 (2) Å
*c* = 13.1339 (3) Å
β = 91.003 (1)°
*V* = 1530.20 (7) Å^3^
*Z* = 4
Mo *K*α radiation
μ = 0.09 mm^−1^
*T* = 100 K
0.42 × 0.36 × 0.18 mm

#### Data collection

Bruker APEXII CCD diffractometer
Absorption correction: multi-scan (*SADABS*; Sheldrick, 2004 ▶) *T*_min_ = 0.962, *T*_max_ = 0.984
26541 measured reflections
3782 independent reflections
3132 reflections with *I* > 2σ(*I*)
*R*_int_ = 0.036

#### Refinement

*R*[*F* ^2^ > 2σ(*F* ^2^)] = 0.042
*wR*(*F* ^2^) = 0.114
*S* = 1.05
3782 reflections
203 parameters
H-atom parameters constrained
Δρ_max_ = 0.31 e Å^−3^
Δρ_min_ = −0.25 e Å^−3^

### 

Data collection: *APEX2* (Bruker, 2008 ▶); cell refinement: *SAINT-Plus* (Bruker, 2008 ▶); data reduction: *SAINT-Plus*; program(s) used to solve structure: *SHELXS97* (Sheldrick, 2008 ▶); program(s) used to refine structure: *SHELXL97* (Sheldrick, 2008 ▶); molecular graphics: *DIAMOND* (Brandenburg & Putz, 2005 ▶); software used to prepare material for publication: *WinGX* (Farrugia, 1999 ▶).

## Supplementary Material

Crystal structure: contains datablock(s) global, I. DOI: 10.1107/S1600536811049865/fk2046sup1.cif

Structure factors: contains datablock(s) I. DOI: 10.1107/S1600536811049865/fk2046Isup2.hkl

Supplementary material file. DOI: 10.1107/S1600536811049865/fk2046Isup3.cml

Additional supplementary materials:  crystallographic information; 3D view; checkCIF report

## Figures and Tables

**Table 1:** Hydrogen-bond geometry (Å, °)

*D*—H⋯*A*	*D*—H	H⋯*A*	*D*⋯*A*	*D*—H⋯*A*
C6—H6*B*⋯O3^i^	0.98	2.42	3.3825 (15)	167
C7—H7*B*⋯O1^ii^	0.98	2.5	3.3826 (15)	149
C13—H13⋯N1^iii^	0.95	2.62	3.2701 (16)	126
C15—H15*A*⋯O2^iv^	0.98	2.56	3.5187 (17)	165

Symmetry codes: (i) 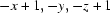; (ii) 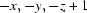; (iii) 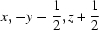; (iv) 
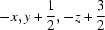
.

**Fig. 1.**
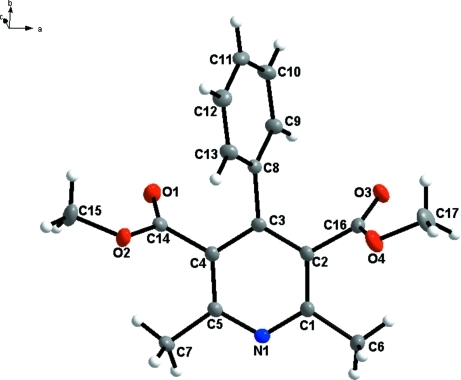
Diamond representation of the title compound, showing the numbering scheme and displacement ellipsoids (50% probability).

## supplementary crystallographic information

### Comment

1,4-Dihydropyridines (1,4-DHPs) belong to a class of nitrogen containing heterocycles having a six-membered ring. These are analogues of NADH coenzymes and are an important class of drugs (Lopez-Alarcon *et al.* 2004). The oxidation of 1,4-DHP`s into the corresponding pyridines is one of the main metabolic pathways of these drugs. The title compound can be prepared by the catalytic oxidation of 1,4-dihydropyridine. The dihydropyrimidine synthesized by the known procedure through three components process disclosed in the literature (Debache *et al.* 2008). The oxidation of the dihdropyridine was carried out in the presence of 5 mol% of I_2_ as a catalyst using DMSO as solvent. The title compound, C_17_H_17_N_1_O_4_, (Figure 1) crystallized in the monoclinic space group P2(1)/c with Z = 4. The dihedral angle between the benzene ring and the pyridine ring is 75.51 (4)°. This compares well to 75.3 (4)° from the structure reported by Lou *et al.* (2010). The benzene ring (C8—C13) is flat (r.m.s = 0.0040) as well as the pyridine (N1, C1—C4) ring (r.m.s =0.0083). So the nitrogen in the pyridine ring is not protonated (Rowan *et al.*, 1996 and 1997). The methyl groups at C1 and C5 are above the plane at 0.0165 (20) Å and 0.0589 (19)Å respectively. The carboxylate groups at C2 and C4 are also out of the plane by 0.0521 (18) and -0.1049 (18) Å respectively. Bond lengths and angles are within expected ranges (Allen *et al.*, 1987). The packing is further stabilized by weak intermolecular C6—H6B···O3^i^, C7—H7B···O1^ii^, C15—H15A···O2^iv^ and C13—H13···N1^iii^ interactions (Table 1). (i = -*x* + 1, -*y*, -*z* + 1; ii = -*x*, -*y*, -*z* + 1; iii = *x*, -*y* - 1/2, *z* + 1/2; iv = -*x*, *y* + 1/2, -*z* + 1.5)

### Experimental

1,4-dihydropyridine synthesis: Methylacetoacetate (2.5 mmol) and benzaldehyde (1 mmol) was added to ethanol (10 ml) and stirred. To this 5 mol % of phenyl boric acid was added as catalyst. The mixture was heated to reflux and stirred until completion. After completion of the reaction (monitored by TLC) the precipitated was filtered off and dried in oven at 60 °C before it was dissolved in a KOH (2 mmol) containing DMSO solution(3 ml). Molecular iodine 5 mol% was added and the mixture stirred at room temperature until completion of the reaction(TLC). Ice cold water (20 ml) was subsequently added and the reaction mixture stirred for 30 min, before the product was extracted into ethyl acetate (3 *x* 20 ml) and the solvent removed under reduced pressure to yield the title compound as a white powder. Crystals suitable for *x*-ray analysis where obtained by slow evaporation of hexane and dicloromethane mixture (9:1; 2 ml) at 4 °C.

^1^H NMR (600 MHz): 2.60 (s, 6H,2 *x* Methyl), 3.54 (s, 6H, 2 *x* methoxy), 7.24 (m, 2H, aromatic-H), 7.39 (m, 3H, aromatic-H).

^13^C NMR (150 MHz): 23.12, 52.35, 126.89, 126.87, 128.38, 128.68, 136.56, 146.41, 155.72, 168.59. m.p. 130–131 °C

### Refinement

H atoms were positioned geometrically and allowed to ride on their parent atoms, with *U*_iso_(H) = 1.2*U*_eq_(C) with a C—H distance of 0.95. The methyl H atoms were derived from difference maps (HFIX 137) and refined with *U*_iso_(H) = 1.5*U*_eq_(C) and C—H 0.98 Å.

### Crystal data

**Table:**

C_17_H_17_NO_4_	*F*(000) = 632
*M**_r_* = 299.32	*D*_x_ = 1.299 Mg m^−^^3^
Monoclinic, *P*2_1_/*c*	Mo *K*α radiation, λ = 0.71073 Å
*a* = 16.0732 (4) Å	Cell parameters from 6764 reflections
*b* = 7.2497 (2) Å	θ = 3.1–28.2°
*c* = 13.1339 (3) Å	µ = 0.09 mm^−^^1^
β = 91.003 (1)°	*T* = 100 K
*V* = 1530.20 (7) Å^3^	Plate, colourless
*Z* = 4	0.42 × 0.36 × 0.18 mm

### Data collection

**Table:**

Bruker APEXII CCD diffractometer	3132 reflections with *I* > 2σ(*I*)
graphite	*R*_int_ = 0.036
φ and ω scans	θ_max_ = 28.3°, θ_min_ = 1.3°
Absorption correction: multi-scan (*SADABS*; Sheldrick, 2004)	*h* = −19→21
*T*_min_ = 0.962, *T*_max_ = 0.984	*k* = −9→9
26541 measured reflections	*l* = −17→16
3782 independent reflections	

### Refinement

**Table:**

Refinement on *F*^2^	Primary atom site location: structure-invariant direct methods
Least-squares matrix: full	Secondary atom site location: difference Fourier map
*R*[*F*^2^ > 2σ(*F*^2^)] = 0.042	Hydrogen site location: geom and difmap
*wR*(*F*^2^) = 0.114	H-atom parameters constrained
*S* = 1.05	*w* = 1/[σ^2^(*F*_o_^2^) + (0.0581*P*)^2^ + 0.5155*P*] where *P* = (*F*_o_^2^ + 2*F*_c_^2^)/3
3782 reflections	(Δ/σ)_max_ = 0.001
203 parameters	Δρ_max_ = 0.31 e Å^−^^3^
0 restraints	Δρ_min_ = −0.25 e Å^−^^3^

### Special details

**Table:**

Experimental. The intensity data was collected on a Bruker X8 ApexII 4 K Kappa CCD diffractometer using an exposure time of 10 s/frame. A total of 1659 frames were collected with a frame width of 0.5° covering up to θ = 28.26° with 99.9% completeness accomplished.
Geometry. All s.u.'s (except the s.u. in the dihedral angle between two l.s. planes) are estimated using the full covariance matrix. The cell s.u.'s are taken into account individually in the estimation of s.u.'s in distances, angles and torsion angles; correlations between s.u.'s in cell parameters are only used when they are defined by crystal symmetry. An approximate (isotropic) treatment of cell s.u.'s is used for estimating s.u.'s involving l.s. planes.
Refinement. Refinement of *F*^2^ against ALL reflections. The weighted *R*-factor *wR* and goodness of fit *S* are based on *F*^2^, conventional *R*-factors *R* are based on *F*, with *F* set to zero for negative *F*^2^. The threshold expression of *F*^2^ > 2σ(*F*^2^) is used only for calculating *R*-factors(gt) *etc*. and is not relevant to the choice of reflections for refinement. *R*-factors based on *F*^2^ are statistically about twice as large as those based on *F*, and *R*- factors based on ALL data will be even larger.

### Fractional atomic coordinates and isotropic or equivalent isotropic displacement parameters (Å^2^)

**Table:**

	*x*	*y*	*z*	*U*_iso_*/*U*_eq_	
C1	0.31920 (7)	−0.26904 (16)	0.51421 (9)	0.0155 (2)	
C2	0.32461 (7)	−0.11146 (15)	0.57614 (9)	0.0143 (2)	
C3	0.25177 (7)	−0.02892 (15)	0.61091 (8)	0.0135 (2)	
C4	0.17589 (7)	−0.11187 (15)	0.58352 (9)	0.0143 (2)	
C5	0.17515 (7)	−0.27106 (16)	0.52304 (9)	0.0151 (2)	
C6	0.39463 (8)	−0.36532 (18)	0.47394 (11)	0.0233 (3)	
H6A	0.4141	−0.4576	0.5234	0.035*	
H6B	0.4388	−0.2748	0.4627	0.035*	
H6C	0.3802	−0.4263	0.4094	0.035*	
C7	0.09654 (8)	−0.37102 (17)	0.49311 (10)	0.0209 (3)	
H7A	0.1002	−0.413	0.4224	0.031*	
H7B	0.049	−0.2875	0.4995	0.031*	
H7C	0.0892	−0.4777	0.5379	0.031*	
C8	0.25268 (7)	0.13135 (15)	0.68224 (9)	0.0140 (2)	
C9	0.27156 (7)	0.30990 (16)	0.65095 (9)	0.0176 (2)	
H9	0.2869	0.3325	0.5826	0.021*	
C10	0.26785 (8)	0.45503 (16)	0.71995 (9)	0.0196 (3)	
H10	0.28	0.5769	0.6982	0.023*	
C11	0.24653 (7)	0.42316 (17)	0.82023 (9)	0.0188 (3)	
H11	0.2441	0.5229	0.867	0.023*	
C12	0.22882 (8)	0.24556 (18)	0.85221 (9)	0.0213 (3)	
H12	0.2148	0.2232	0.9211	0.026*	
C13	0.23166 (8)	0.10024 (17)	0.78347 (9)	0.0188 (3)	
H13	0.2192	−0.0213	0.8055	0.023*	
C14	0.09598 (7)	−0.02242 (16)	0.61348 (9)	0.0168 (2)	
C15	−0.02859 (8)	−0.0577 (2)	0.70350 (13)	0.0324 (3)	
H15A	−0.0238	0.0649	0.7345	0.049*	
H15B	−0.0548	−0.1425	0.7514	0.049*	
H15C	−0.0627	−0.0498	0.6411	0.049*	
C16	0.40794 (7)	−0.03409 (16)	0.60534 (9)	0.0163 (2)	
C17	0.53414 (8)	−0.0953 (2)	0.69408 (12)	0.0314 (3)	
H17A	0.568	−0.0971	0.6327	0.047*	
H17B	0.5573	−0.1822	0.7442	0.047*	
H17C	0.5344	0.0293	0.723	0.047*	
N1	0.24576 (6)	−0.34462 (13)	0.48819 (7)	0.0154 (2)	
O1	0.07254 (6)	0.12423 (12)	0.58103 (8)	0.0250 (2)	
O2	0.05370 (6)	−0.12553 (12)	0.67903 (7)	0.0227 (2)	
O3	0.43443 (6)	0.11142 (12)	0.57660 (8)	0.0236 (2)	
O4	0.44906 (6)	−0.14856 (13)	0.66776 (7)	0.0255 (2)	

### Atomic displacement parameters (Å^2^)

**Table:**

	*U*^11^	*U*^22^	*U*^33^	*U*^12^	*U*^13^	*U*^23^
C1	0.0148 (5)	0.0143 (5)	0.0174 (5)	−0.0007 (4)	0.0027 (4)	0.0020 (4)
C2	0.0138 (5)	0.0132 (5)	0.0159 (5)	−0.0015 (4)	0.0001 (4)	0.0024 (4)
C3	0.0154 (5)	0.0111 (5)	0.0139 (5)	−0.0003 (4)	0.0003 (4)	0.0026 (4)
C4	0.0141 (5)	0.0131 (5)	0.0158 (5)	0.0002 (4)	0.0002 (4)	0.0020 (4)
C5	0.0150 (5)	0.0136 (5)	0.0166 (5)	−0.0012 (4)	−0.0008 (4)	0.0017 (4)
C6	0.0168 (6)	0.0202 (6)	0.0331 (7)	−0.0010 (5)	0.0066 (5)	−0.0062 (5)
C7	0.0157 (6)	0.0183 (6)	0.0286 (7)	−0.0023 (5)	−0.0019 (5)	−0.0055 (5)
C8	0.0126 (5)	0.0137 (5)	0.0157 (5)	−0.0002 (4)	−0.0015 (4)	−0.0002 (4)
C9	0.0213 (6)	0.0154 (6)	0.0162 (5)	−0.0018 (4)	0.0009 (4)	0.0014 (4)
C10	0.0232 (6)	0.0131 (5)	0.0223 (6)	−0.0020 (5)	−0.0023 (5)	0.0001 (5)
C11	0.0183 (6)	0.0181 (6)	0.0197 (6)	0.0012 (4)	−0.0024 (5)	−0.0056 (4)
C12	0.0261 (6)	0.0228 (6)	0.0151 (5)	−0.0013 (5)	0.0012 (5)	−0.0001 (5)
C13	0.0232 (6)	0.0153 (5)	0.0179 (6)	−0.0022 (5)	0.0001 (5)	0.0024 (4)
C14	0.0142 (5)	0.0155 (5)	0.0207 (6)	−0.0016 (4)	−0.0015 (4)	−0.0030 (4)
C15	0.0200 (7)	0.0269 (7)	0.0508 (9)	0.0028 (5)	0.0155 (6)	0.0014 (6)
C16	0.0135 (5)	0.0167 (6)	0.0189 (6)	0.0001 (4)	0.0020 (4)	−0.0020 (4)
C17	0.0180 (6)	0.0337 (8)	0.0422 (8)	−0.0033 (5)	−0.0109 (6)	0.0044 (6)
N1	0.0169 (5)	0.0133 (5)	0.0160 (5)	−0.0006 (4)	0.0007 (4)	0.0009 (4)
O1	0.0198 (5)	0.0183 (4)	0.0368 (5)	0.0049 (3)	0.0015 (4)	0.0042 (4)
O2	0.0173 (4)	0.0203 (4)	0.0308 (5)	0.0013 (3)	0.0084 (4)	0.0020 (4)
O3	0.0174 (4)	0.0173 (4)	0.0361 (5)	−0.0043 (3)	0.0001 (4)	0.0031 (4)
O4	0.0178 (4)	0.0254 (5)	0.0330 (5)	−0.0043 (4)	−0.0084 (4)	0.0081 (4)

### Geometric parameters (Å, °)

**Table:**

C1—N1	1.3402 (15)	C9—H9	0.95
C1—C2	1.4042 (16)	C10—C11	1.3861 (17)
C1—C6	1.5032 (16)	C10—H10	0.95
C2—C3	1.3986 (15)	C11—C12	1.3854 (18)
C2—C16	1.4958 (16)	C11—H11	0.95
C3—C4	1.4011 (15)	C12—C13	1.3887 (17)
C3—C8	1.4924 (15)	C12—H12	0.95
C4—C5	1.4009 (16)	C13—H13	0.95
C4—C14	1.4977 (16)	C14—O1	1.2034 (15)
C5—N1	1.3417 (15)	C14—O2	1.3350 (15)
C5—C7	1.5030 (16)	C15—O2	1.4524 (15)
C6—H6A	0.98	C15—H15A	0.98
C6—H6B	0.98	C15—H15B	0.98
C6—H6C	0.98	C15—H15C	0.98
C7—H7A	0.98	C16—O3	1.2009 (15)
C7—H7B	0.98	C16—O4	1.3338 (15)
C7—H7C	0.98	C17—O4	1.4568 (15)
C8—C9	1.3932 (16)	C17—H17A	0.98
C8—C13	1.3959 (16)	C17—H17B	0.98
C9—C10	1.3906 (17)	C17—H17C	0.98
			
N1—C1—C2	121.72 (10)	C11—C10—C9	120.48 (11)
N1—C1—C6	115.63 (10)	C11—C10—H10	119.8
C2—C1—C6	122.64 (11)	C9—C10—H10	119.8
C3—C2—C1	119.57 (10)	C12—C11—C10	119.90 (11)
C3—C2—C16	120.45 (10)	C12—C11—H11	120
C1—C2—C16	119.98 (10)	C10—C11—H11	120
C2—C3—C4	117.54 (10)	C11—C12—C13	119.92 (11)
C2—C3—C8	122.61 (10)	C11—C12—H12	120
C4—C3—C8	119.62 (10)	C13—C12—H12	120
C5—C4—C3	119.86 (10)	C12—C13—C8	120.52 (11)
C5—C4—C14	120.45 (10)	C12—C13—H13	119.7
C3—C4—C14	119.57 (10)	C8—C13—H13	119.7
N1—C5—C4	121.47 (10)	O1—C14—O2	124.29 (11)
N1—C5—C7	115.56 (10)	O1—C14—C4	123.65 (11)
C4—C5—C7	122.97 (10)	O2—C14—C4	112.06 (10)
C1—C6—H6A	109.5	O2—C15—H15A	109.5
C1—C6—H6B	109.5	O2—C15—H15B	109.5
H6A—C6—H6B	109.5	H15A—C15—H15B	109.5
C1—C6—H6C	109.5	O2—C15—H15C	109.5
H6A—C6—H6C	109.5	H15A—C15—H15C	109.5
H6B—C6—H6C	109.5	H15B—C15—H15C	109.5
C5—C7—H7A	109.5	O3—C16—O4	124.40 (11)
C5—C7—H7B	109.5	O3—C16—C2	124.73 (11)
H7A—C7—H7B	109.5	O4—C16—C2	110.87 (10)
C5—C7—H7C	109.5	O4—C17—H17A	109.5
H7A—C7—H7C	109.5	O4—C17—H17B	109.5
H7B—C7—H7C	109.5	H17A—C17—H17B	109.5
C9—C8—C13	119.26 (11)	O4—C17—H17C	109.5
C9—C8—C3	122.55 (10)	H17A—C17—H17C	109.5
C13—C8—C3	118.18 (10)	H17B—C17—H17C	109.5
C10—C9—C8	119.91 (11)	C1—N1—C5	119.80 (10)
C10—C9—H9	120	C14—O2—C15	115.40 (10)
C8—C9—H9	120	C16—O4—C17	115.72 (10)

### Hydrogen-bond geometry (Å, °)

**Table:**

*D*—H···*A*	*D*—H	H···*A*	*D*···*A*	*D*—H···*A*
C6—H6B···O3^i^	0.98	2.42	3.3825 (15)	167.
C7—H7B···O1^ii^	0.98	2.5	3.3826 (15)	149.
C13—H13···N1^iii^	0.95	2.62	3.2701 (16)	126.
C15—H15A···O2^iv^	0.98	2.56	3.5187 (17)	165.

Symmetry codes: (i) −*x*+1, −*y*, −*z*+1; (ii) −*x*, −*y*, −*z*+1; (iii) *x*, −*y*−1/2, *z*+1/2; (iv) −*x*, *y*+1/2, −*z*+3/2.
